# Gastric Electrical Stimulation with the Enterra System: A Systematic Review

**DOI:** 10.1155/2015/762972

**Published:** 2015-07-12

**Authors:** Nikhil Lal, Sam Livemore, Declan Dunne, Iftikhar Khan

**Affiliations:** ^1^School of Medicine, University of Liverpool, Liverpool L69 3GE, UK; ^2^Aintree University Hospital, Lower Lane, Fazakerley, Liverpool L9 7AL, UK

## Abstract

*Background*. Gastric electrical stimulation (GES) is a surgically implanted treatment option for refractory gastroparesis. *Aim*. To systematically appraise the current evidence for the use of gastric electrical stimulation and suggest a method of standardisation of assessment and follow-up in these patients. *Methods*. A systematic review of PubMed, Web of Science, DISCOVER, and Cochrane Library was conducted using the keywords including gastric electrical stimulation, gastroparesis, nausea, and vomiting and neuromodulation, stomach, central nervous system, gastric pacing, electrical stimulation, and gastrointestinal. *Results*. 1139 potentially relevant articles were identified, of which 21 met the inclusion criteria and were included. The quality of studies was variable. There was a variation in outcome measures and follow-up methodology. Included studies suggested significant reductions in symptom severity reporting over the study period, but improvements in gastric emptying time were variable and rarely correlated with symptom improvement. *Conclusion*. The evidence in support of gastric electrical stimulation is limited and heterogeneous in quality. While current evidence has shown a degree of efficacy in these patients, high-quality, large clinical trials are needed to establish the efficacy of this therapy and to identify the patients for whom this therapy is inappropriate. A consensus view on essential preoperative assessment and postoperative measurement is needed.

## 1. Introduction

Gastric electrical stimulation (GES) is a surgically implanted treatment option for treating gastroparesis resistant to medical therapy. Gastroparesis is characterised by a delay in gastric emptying in the absence of any physical obstruction [1, 2]. Patients who suffer from gastroparesis often report a significant reduction in their quality of life [3]. The cornerstone of treatment is symptomatic medical management including dietary modification, prokinetics, and nutritional supplementation [4, 5]. Timing of prokinetic administration to ensure a high bioavailability of the prokinetic is an important consideration [6]. Bortolotti et al. [6] observed a substantial improvement in dyspeptic symptoms in patients after administration of prokinetics 2.5 hours before meals [6]. This is a key consideration in the management of refractory gastroparesis and limits the need for further intervention [6]. Despite optimal medical therapy, patients with refractory gastroparesis require frequent hospitalizations. This impairs quality of life, and incurs considerable cost to the health services [7]. Patients with gastroparesis refractory to medical management can be considered for gastric electrical stimulation or gastric pacing.

Three principal methods are currently available: gastric low-frequency/high-energy GES with long pulse stimulation, high-frequency/low-energy GES with short pulse stimulation, and neural sequential GES [8]. Neural sequential GES is not used in humans currently [8]. Low-frequency/high-energy GES involves heavy batteries and is not suitable for implantation; it also has a variable effect on the symptoms [8]. High-frequency/low-energy GES, also known as the Enterra (Medtronic, Minneapolis, USA) Therapy, improves dyspeptic symptoms, such as nausea and vomiting, giving patients a better quality of life together with a more satisfactory nutritional status, and is suitable for implantation.

Insertion of GES device involves the surgical placement of several electrodes into the muscle layer of the stomach, typically delivering two short pulses with an interval of 72 ms, width of about 0.3 ms, and amplitude of about 5 mA7 [9, 10]. In the United States of America the Food and Drug Administration (FDA) approval was given through a “humanitarian device exemption,” a regulatory category established in 1996 applying to interventions intended to benefit less than 4000 patients [11]. Recent studies have suggested an improvement in the quality of life or symptoms or both for patients undergoing GES [9, 10, 12–14], though controversy exists with concern about the standard of studies into the efficacy of this therapy. There is particular concern about the absence of control groups [9, 14–16]. Recent studies regarding the effectiveness of gastric electrical stimulation have shown variability in the outcome measures used for pre- and post-op assessment of the patients [9, 10, 17–19]. This review aimed to systematically appraise the current evidence for the use of gastric electrical stimulation and suggest a method of standardisation of assessment outcome reporting.

## 2. Methods

A review protocol was devised regarding search strategy and data extraction. Studies published since 1993 were identified using PubMed, Web of Knowledge, DISCOVER, and the Cochrane Library. The search terms included those used originally by Zhang and Chen (2006) [20], including the following additional keywords: “gastroparesis,” “gastric electrical stimulation,” “neuromodulation,” “central nervous system,” “gastrointestinal,” “nausea and vomiting,” “gastric pacing,” and “stomach.” The Enterra (Medtronic, Minneapolis, USA) device is the most widely utilized device and has been the focus of this review. Non-English publications and nonhuman studies were excluded from the results. Search results were examined with regard to title and abstract and suitable studies identified. Included studies had to be primary research, involving at least ten participants with a minimum of six months of follow-up, specifically evaluating the outcomes of permanent gastric electrical stimulation in the treatment of diagnosed gastroparesis. This included randomised controlled trials and open-label cohort and case-control studies. Included participants were required to be at least 18 years of age. This systematic review is reported in accordance with Preferred Reporting Items for Systematic Reviews and Meta-Analyses (PRISMA) guidelines [21].

### 2.1. Data Synthesis and Analysis

Data were extracted from reports by the authors using a preprepared spreadsheet. The data extracted was as follows: study year, sample size, participant demographics and aetiology of gastroparesis, study design and methods, follow-up duration, and outcome measures: symptom scores, gastric emptying time, nutritional status, quality of life, medication usage, weight, and BMI. Risk of bias was assessed using Cochrane Review guidelines [22]. Quality of included trials was assessed using “Consolidated Standards of Reporting Trials” (CONSORT) guidelines, while nontrial studies were assessed using the NICE guidelines for public health intervention research [12, 21].

## 3. Results

**Figure 1:**
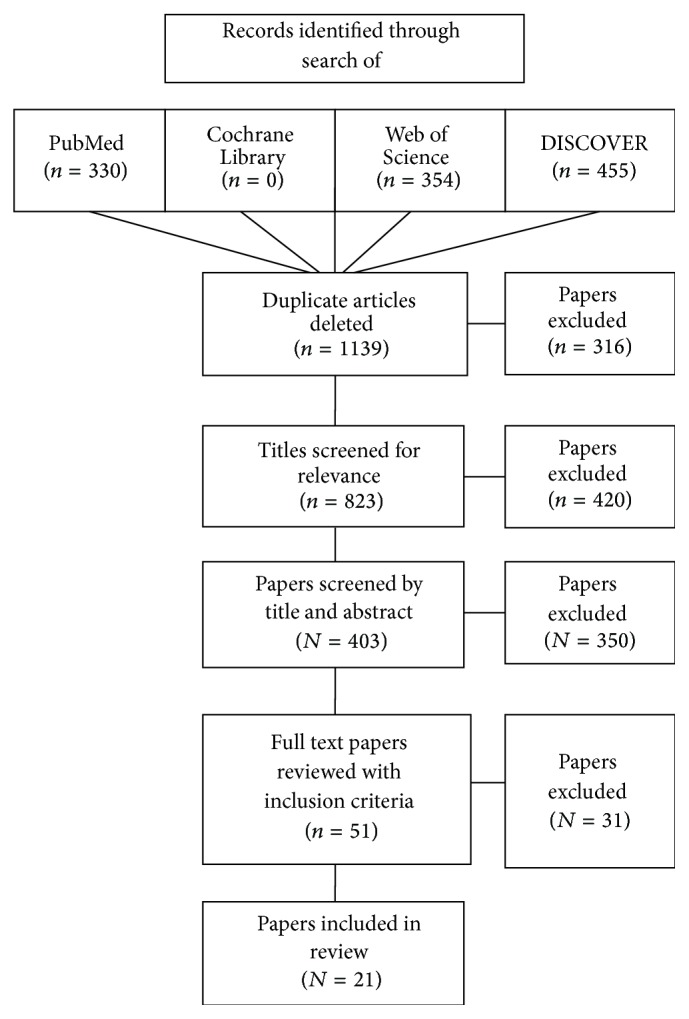
Flow chart of paper selection and analysis.

Through database searching of PubMed, DISCOVER, Web of Knowledge, and the Cochrane Library 1139 papers were identified. Of these, 52 were deemed potentially relevant based on assessment of their title and abstract. After removal of reviews, small sample-sized studies, and irrelevant papers as judged by full text review (Figure 1), the final review consisted of 21 studies. 12 of these studies were conducted by a study group involving RW McCallum. The characteristics of the included studies are shown in Tables 1 and 2 and included 3 crossover studies and 18 prospective cohort studies. The three crossover studies became observational in nature after several months.

### 3.1. Quality of Trials and Risk of Bias

Overall the risk of bias was considered medium to high in the majority of studies with low risk being suggested only in Abell et al., 2003 [22]. The main reason was the frequency of nonrandomised trials which under Cochrane Review guidelines are deemed to be at a higher risk of bias.

In addition it was noted that six of the studies used the same hospital and time period (Kansas University Medical Centre between 1998 and 2002) [10, 22–25]. Many patients were also enrolled on several large scale studies concurrently, for example, WAVESS [22], GEMS [10], and CUESS (CUESS is referred to many times in the literature, but there are no published journal articles that are specifically and explicitly entitled or identified as the “Compassionate Electrical Simulation Study.” It is apparently in reference to Forster et al., 2001 [9]. WAVESS: Worldwide Antivomiting Electrical Stimulation Study; GEMS: Gastric Electromechanical Stimulation; CUESS: Compassionate Use of Electrical Stimulation Study).

### 3.2. Symptom Scores

There is a variation in the methods used to assess the improvement in symptoms in the patients with GES implants. Most commonly used measures were Total Symptom Score (TSS) [12, 14, 15, 19, 23–29], Gastroparesis Cardinal Symptom Index (GCSI) [30], Weekly Vomiting Frequency [10, 16, 18, 19, 22, 28], Weekly Nausea Frequency [10, 18, 22, 28], Monthly Vomiting Frequency [10], Monthly Nausea Frequency [31], and Gastrointestinal Symptoms Rating Scale (GSRS) [32].

To date there have been just three studies fulfilling employing a blinded period of study [16, 22]. In a 2003 study (Abell et al., 2003) [22], 33 patients (17 diabetic, 16 idiopathic) underwent a one-month, blinded crossover condition whereby they experienced a one-month ON period followed by a one-month OFF period. The crossover period (phase I) was followed by a 12-month open-label phase with regular follow-ups (phase II). In phase I, significant improvement in median vomiting frequency was found in the ON period in the combined (all patients) group, although the same measure in specific patient groups (diabetic and idiopathic) did not alter significantly between conditions. Patient preference for ON treatment compared to OFF treatment was significant in the combined and idiopathic groups, but not the diabetic patients. At 12 months, Weekly Vomiting Frequency had decreased by over 60% in both groups of patients, with a greater than 80% reduction seen in half of the patients involved in the study [22].

In the second example, McCallum et al., 2010 [16], a study of 55 diabetic patients inserted with GES, reported significant improvements in symptom reporting following the surgery. Patients underwent randomised 3-month ON or OFF period, followed by 3 months of the reverse condition. This was concluded with 4.5 months of ON condition up to a 12-month follow-up. There were no significant improvements shown in the initial crossover period. However, by 12 months Weekly Vomiting Frequency had significantly declined from a mean of 19.5 to just 4.25 episodes per week (a 78% reduction, *p* < 0.005), while Total Symptom Scores (TSS) were reduced in terms of frequency and severity by 35% and 37%, respectively [16].

Additionally, several large scale nonrandomised studies have displayed similar results in terms of symptom improvements. Most notably, McCallum et al., 2011 [29], in a study of 188 patients, found TSS to improve from 19.4 to 9.2 (52.6% here, with *p* < 0.001) [29]. Anand et al., 2007 [28], found similar improvements in TSS (30.1% or 15.6 to 10.9, *p* < 0.001) in a study of 214 patients over a median follow-up of 4 years. In a recent study involving 32 patients, Mccallum et al., 2013 [19], reported a significant reduction in TSS frequency and severity scores (*p* < 0.001). Brody et al., 2015 [14], also noted a significant reduction in TSS frequency and pain scores for 79 patients.

### 3.3. Gastric Emptying

All studies investigating gastric emptying used a 2-hour and 4-hour Gastric Emptying Test (GET) after a low fat meal. Seven studies [13, 19, 22, 28, 29, 33, 34] noted a significant improvement in GE. On the other hand, seven studies [9, 10, 12, 15, 17, 23, 27] noted no significant change in GE. It should be noted that in a handful of studies [17, 33] patients continued to consume prokinetics during the study period, which represents a confounded potential of the results. Only one study, O'Loughlin et al., 2013 [30], reported a significant correlation between changes in gastric emptying and improvements in symptom reporting, finding a correlation coefficient of 0.693 (*p* = 0.0086) in a study of 14 patients [30]. Other studies, such as Brody et al., 2008 [13], found that symptom severity was reduced in all patients with normal gastric emptying postoperatively [13].

Abell et al. (2003) [22] displayed significant improvements as a result of GES insertion. Two- and 4-hour gastric emptying improved significantly at 6 and 12 months for the combined group, with 2-hour retention falling from 78% to 56% at 12 months (*p* < 0.05). Diabetic patients also saw a significant decline in gastric retention at 4 hours, falling from 46 to 16% at 12 months (*p* < 0.05). However, the study concluded that no correlation existed between changes in vomiting frequency and improvements in gastric emptying time [22]. Similar results were seen elsewhere (McKenna et al., 2008 [15]), where, in a study of 19 patients, they found significant improvements in gastric emptying at 6-month follow-up but did not find a significant difference in symptom reporting between patients who had their gastric emptying times normalised and those in whom it was still delayed [15]. Mccallum et al., 2013 [19], observed no significant reduction in gastric emptying. The 2 hr and 4 hr retention fell from 63.5%  and 26% to 4.9%  and 16.5%, respectively, during the 12-month follow-up [19].

Pyloroplasty was only rarely carried out in conjunction with insertion of GES. Only 4 (0.73%) patients out of 545 patients who were reviewed for GE in the studies included in the review had pyloroplasty during the assessment. From the 3 studies that included patients with pyloroplasty, only Mason et al. [33] noted a significant change in GE.

### 3.4. Quality of Life

Papers reported an increase in quality of life as analysed mainly via Short Form 36 (SF-36) [15–19, 22, 23, 26, 27] or an Independent Outcome Measure System (IDIOMS) [28]. The major areas of improvement were seen in the physical and mental components. Abell et al. displayed a significant postoperative increase of 40.7% and 24.6% (*p* < 0.05) in the physical and mental component scores, respectively, in a 2004 follow-up of 28 patients [26]. A few studies [16, 19] also noted a significant increase in the quality of life. However, McKenna el al., 2008 [15], found the opposite with no significant increase in the quality of life.

### 3.5. Hospital Admissions, Medication Requirements, and Nutritional Status

The number of days spent in the hospital in the year after GES is commenced is reported to be between 13 and 31% of the number spent in the year before the surgery [16, 17, 23–25, 29]. Lin et al., 2005 [24], found a significant reduction in the postoperative use of prokinetics and antiemetics. Patients who no longer require medications, such as prokinetics and antiemetics, postoperatively show a significantly better outcome than those patients who still require medications after undergoing GES [24]. Significant reductions were also displayed in the nutritional support required by these patients postoperatively [9, 10, 17, 22, 33]. In a 2002 study by Abell et al. [10], of the 11 patients requiring enteral nutrition at baseline, just three still required this intervention at 12 months [10].

### 3.6. Survival

None of the included studies reported a death associated with GES implantation. One study analysed long-term survival in implanted patients. Anand et al., 2007 [28], a study of 214 patients, found no difference in survival at 30 months between patients who underwent GES and those who did not. However, significantly poorer 30-month survival rates were displayed in diabetic patients (85%) when compared to their idiopathic counterparts (91.1%). The poorest outcome was shown to be in diabetic patients who did not undergo GES [28].

### 3.7. Adverse Events

Adverse events can be noted in patients after GES insertion, although serious adverse events such as migration of the leads and infections of the pocket of the pulse generator are infrequent. Typically complications occur between 5 and 14% [16, 17, 19, 22, 23]. Abell et al. [22] found that 3 of 33 patients (9.1%) with idiopathic GP participants suffered from infection of the pulse generator site, migration and erosion of the stimulating device [22]. All these complications require surgical intervention and are detrimental to the patient's health. Lin et al., 2004 [23], noted that 8.3% of the patients suffered from infection at the pulse generator site. McCallum et al. [17] noted that only 2 (12.5%) patients had serious adverse events and one patient suffered from infection in the pocket of the pulse generator, while another patient had a dislodged lead. In McCallum et al. [16] 3 (5.45%) out of 55 patients suffered from lead migration/dislodgements, 2 device migrations, 1 implant site hematoma, and 1 implant site infection. Mccallum et al. [19] observed that only 14.1% of the adverse events were linked to the therapy. Out of these the serious adverse events involved a dislodged lead, infection, and paraesthesia. Mason et al. [33] reported no adverse events apart from infections following surgery for GES insertion. However Brody at al. [14] and Ross et al. [32] also reported no adverse events.

## 4. Discussion

These studies included in the review used a variety of outcome measures and variety of preoperative assessments, making it difficult to combine data and offer firm conclusions. The evidence base for the use of GES in gastroparesis is limited with a total of just five months of blinded, randomised study including only 83 patients [9, 10, 12, 13, 15–18, 23–30, 33]. However, accepting the limitations of the evidence base, the majority of studies reported an improvement in symptomology and quality of life with GES [9, 10, 12, 13, 15–19, 22–30, 33]. An improvement in gastric emptying was seen in most studies, with only two failing to demonstrate an improvement [10, 23]. However with the exception of Mccallum et al., 2013 [19], improved gastric emptying did not correlate with the improved symptomology.

The absence of a standardised approach to symptom reporting and preoperative assessment means comparison between studies is challenging, and combining results in a meta-analysis is not possible. The commonest symptom assessments used include Total Symptom Score (TSS) and Gastroparesis Cardinal Symptom Index (GCSI) for scoring symptoms. Some solely measured weekly vomiting and nausea frequency. For example, Abell et al., 2002 [10], used Monthly Vomiting Frequency (MVF), Monthly Nausea Frequency (MNF), Weekly Vomiting Frequency (WVF), and Weekly Nausea Frequency (WNF) as a measure of the improvement in the patient's symptoms. On the other hand, studies such as McCallum et al., 2005 [17], used Total Symptom Score (TSS) as a measure for symptoms. Quality of life was measured using either the Short Form 36 (SF-36) or the Health-Related Quality of Life Score.

Gastric emptying was mainly measured using standardised scintigraphy analysis of retention of an isotope labelled meal. There was a discrepancy in the definition of delayed gastric emptying employed in the included studies [29].

Only two studies suggested a correlation between gastric emptying and symptoms [24, 30]. This questions our understanding of the underlying mechanism that contributes the symptoms of gastroparesis. Since improvements in gastric emptying correlate poorly with changes in patient symptom reporting, perhaps other mechanisms underpin this. Zhang and Chen [20] suggested that GES did not alter or improve gastric emptying and that symptom improvement might be attributed to the improvements in gastroparetic symptoms and overall clinical profile of the patients. Hou et al., 2012 [35], noted that diabetic patients were more likely to have a correlation between symptom relief and GR reduction. This warrants further investigation. A standardised method for testing gastric emptying and a standardised reporting would aid future research.

McCallum et al., 2011 [29], reported that diabetic and postsurgical gastroparetic patients achieved a greater degree of improvement versus idiopathic gastroparetic patients. This could be because idiopathic gastroparetic patients tend to have a multifactorial cause for their refractory symptoms. Meanwhile, Brody et al., 2015 [14], reported that TSS were decreased significantly for diabetic and idiopathic patients. Improvement in symptoms not only was persistent over time, but also actually seemed to improve gradually during long-term follow-up. This warrants investigation with larger study groups.

The crossover trials offer conflicting views. While Abell et al., 2003 [22], found significant differences between the ON and OFF condition, this was isolated to a single symptom measure and did not comply across individual patient groups. McCallum et al., 2010 [16], did not display any significant results in the three-month blinded crossover trial. Significance in improvements in both instances was seen at 12 months, but this raises the question of why there is heterogeneity in the outcomes even within the higher quality studies. This suggests either the presence of other variables that contribute to the symptom reporting in the long term or that short-term symptom reporting does not correlate with future outcome. Without more, long-term blinded studies, these points cannot be clarified. Assessing the placebo effect is difficult given that subjecting patients to the implantation risks without turning on the device could be ethically questioned. In studies with an OFF phase early differences in symptoms are potentially confounded by the recovery period after surgery, alterations to pain medications and glucose control, and the placebo effect of the surgery itself [16]. However this has suggested GES has an effect above placebo [16, 22]. Brody et al., 2015 [14], argued that no placebo effect could last for more than few weeks and sustain a significant decrease in TSS for these patients for more than 12 months.

Patient study populations also pose significant confounding within the literature. Many of the study patients were recruited at the same study centre during the same time period (Kansas University Medical Centre between 1998 and 2003) [10, 12, 17, 23–25, 27, 28] and many patients were enrolled on several large scale studies simultaneously [9, 10, 22]. This could mean that similar results between studies could reflect the same patients being represented multiple times within the literature. Given that few studies included over 20 patients, the relevance of the studied population in these small studies may be questioned, especially given the known individual variability of the condition [9, 10, 12–14, 16, 19, 24, 26–30, 32].

Follow-up reporting was not consistent, and many papers did not state mean or median follow-up time or at which stage of follow-up the results presented reflected. In one instance a mean follow-up of 56 months referred to a range of 12 to 123 months [29]. This point is of particular importance when considered alongside the level of attrition seen in many of the included studies.

A dominant financial support within the literature was Medtronic (Minneapolis, USA), with 14 of the 21 studies included in this review reported being financially supported, either in part or in full, by it [9, 12, 18, 22, 23, 25–27, 29]. In one instance Medtronic was also responsible for statistical analysis [16]. Given that this is the company that produces the Enterra GES apparatus this represents a potential conflict of interests and could imply a reduced level of impartiality and degree of bias in the included literature. Of the 21 articles selected, 12 featured a study group involving McCallum and 5 featured Abell. It is possible that the publications of each group included a series of patients previously treated that may have led to a bias.

### 4.1. Implications for Further Research and Future Practice

Given the absence of high-quality data to support the efficacy of GES for gastroparesis, there is a need for a randomised crossover study, which based on the safety record of the intervention and analysis of long-term survival in patients postoperatively [28] would be fully justified. The methodology utilised by Abell et al. (2003) [22] and McCallum et al. (2005) [17] would help in determining an approach.

A better understanding of preoperative patient factors that contribute to outcome is needed to improve selection of patients for this therapy and consequently outcomes. GES does appear to offer a significant benefit to a subset of patients, and future research should be aimed at identifying this subset preoperatively. Limited research into this has suggested potential predictive factors that need further exploration [36, 37].

Investigation into novel surgical approaches could include the addition of pyloroplasty during GES insertion which was suggested to improve gastric emptying when compared to patients who were given GES alone (64% GES + PP improvement versus 7% GES, *p* < 0.001) [38]. However, pyloroplasty is associated with significant adverse events such as dumping syndrome, and given the poor correlation with gastric emptying and quality of life the authors would not advocate this approach [38]. Additionally, temporary or transoesophageal GES has been investigated as a potential method for a less invasive trial prior to permanent GES insertion, with mixed results [34, 39]. A final point of note is that the device has many potential settings, including adjustments to frequency, time spent ON or OFF, and Hertz. An algorithm for the application of these factors in setting the device is another possible area of future study. A 2006 study into this area highlighted the benefit of targeted settings in different patient categories [31].

## 5. Conclusion

Gastroparesis has a significant impact on a patient's quality of life and is associated with significant economic cost. Although its supporting evidence base is limited, GES does seem to offer significant improvement in quality of life and symptom control to a subset of patients. The limited current use of this intervention lends itself to easily implementable strategy for the improvement of data quality. To facilitate improved understanding of GES and who it may benefit, an international registry, with standardised preoperative assessment (Table 3), and standardised reporting of outcomes should be introduced.

## Figures and Tables

**Table 1:** Summary of studies.

Study	Methods	*N*	Participants	Follow-up
Forster et al., 2001 [9]	Cohort-observational	25	19 DG, 3 IG, and 3 PSG	12 months
Abell et al., 2002 [10]	Cohort-observational	38	9 DG, 24 IG, and 5 PSG	11 months
Abell et al., 2003 [22]	Crossover and then observational	33	17 DG, 16 IG	12 months
Abell et al., 2003 [26]	Cohort-observational	12	3 DG, 9 IG	60 months
Forster et al., 2003 [27]	Cohort-observational	55	39 DG, 7 IG, and 9 PSG	12 months
Lin et al., 2004 [23]	Cohort-observational	48	48 DG	12 months
McCallum et al., 2005 [17]	Cohort-observational	16	16 PSG	12 months
van der Voort et al., 2005 [18]	Cohort-observational	17	17 DG	12 months
Lin et al., 2005 [24]	Cohort-observational	37	24 DG, 8 IG, and 5 PSG	12 months
Mason et al., 2005 [33]	Cohort-observational	29	24 DG, 5 IG	20 months
Lin et al., 2006 [25]	Cohort-observational	55	39 DG, 9 IG, and 6 PSG	36 months
Anand et al., 2007 [28]	Cohort-observational	214	146 IG, 45 DG, and 23 PSG	48 months
Lin et al., 2008 [12]	Cohort-observational	63	38 DG, 11 IG, and 14 PSG	12 months
McKenna et al., 2008 [15]	Cohort-observational	19	10 DG, 6 IG, and 3 PSG	9.5 months
Brody et al., 2008 [13]	Cohort-observational	50	20 DG, 25 IG, 2 PSG, and 3 CTG	12 months
McCallum et al., 2010 [16]	Crossover and then observational	55	55 DG	12 months
McCallum et al., 2011 [29]	Cohort-observational	221	142 DG, 48 IG, and 31 PSG	56 months
O'Loughlin et al., 2013 [30]	Cohort-observational	17	9 DG, 7 IG, and 1 PSG	14 months
McCallum et al., 2013 [19]	Crossover and then observational	32	32 IG	12 months
Ross et al., 2014 [32]	Cohort-observational	25	15 DG, 10 IG	6 months
Brody et al., 2015 [14]	Cohort-observational	79	43 IG, 37 DG	12 months

DG: diabetic gastroparesis; IG: idiopathic gastroparesis; PSG: postsurgical gastroparesis; WVF: Weekly Vomiting Frequency; WNF: Weekly Nausea Frequency; GET: gastric emptying; SAQ: self-administered questionnaire; M: months; Y: years; W: weeks; TSS: Total Symptom Score; PCS: Physical Composite Score; MCS: Mental Composite Score (PCS and MCS are aspects of QOL assessment); EFT: enteral feeding tube; TPN: total parenteral nutrition; N: nausea; V: vomiting; PK: prokinetics; AE: antiemetics; PPF: postprandial fullness; ILM: isotope labelled meal.

**Table 2:** Study characteristics.

Study	Follow-up	Measure	Outcome at 12 months (unless otherwise stated)
Forster et al., 2001 [9]	12 M	Symptoms	Significant improvement in N and V
		GE	No significant improvement

Abell et al., 2002 [10]	3 M	Symptoms	MVF: 21 to 0; MNF: 21 to 2
		GE	No significant improvement
	12 M	Symptoms	WVF down to average 90%; WNF: 28 to 1
		GE	No significant improvement reported
		Medication	Significant decrease in use of AE/PK; patients requiring none rose 5 to 14

Abell et al., 2003 [22]	2 M	Symptoms	WVF in combined group (all patients) significantly reduced
	12 M	Symptoms	WNF down to 64% baseline in combined group
		GE	78% to 56% 2-hour retention of ILM (*p* < 0.05); 46% to 16% 4-hour retention of ILM (*p* < 0.05)
		Quality of life	PCS: 25.8 to 32.4; MCS: 36.1 to 45.1 (combined group)
		Nutrition	9 out of 14 discontinued nutrition

Abell et al., 2003 [26]	60 M	Symptoms	Mean TSS scores: 35.6 to 16.6; at 60 M TSS score mean at 20.3 (*p* < 0.01), WVS: 3.9 to 1.4 (*p* < 0.01)
		Quality of life	Overall score increased by mean 2.1 points by 60 M

Forster et al., 2003 [27]	12 M	Symptoms	TSS severity mean: 20 to 9.1; frequency mean: 21 to 10
		Quality of life	MCS: 37 to 48; PCS: 24 to 33
		GE	No change
		BMI	BMI and body weight increased significantly

Lin et al., 2004 [23]	12 M	Symptoms	TSS severity mean: 17.6 to 7.9; frequency mean: 18.5 to 8.9 (*p* < 0.05)
		GE	No significant change (significance seen at 6 M)
		Quality of life	MCS: 36.9 to 46; PCS: 23.8 to 33.5 (*p* < 0.05)
		Days in hospital	Mean hospital stay reduced by 52 days compared to the prior year

McCallum et al., 2005 [17]	12 M	Symptoms	TSS severity: 17.1 to 8.6; frequency: 19.2 to 9.89 (*p* < 0.05)
		GE	No significant change
		Quality of life	PCS: 28.6 to 37.7; MCS: 39.7 to 49.6 (*p* < 0.05)
		Days in hospital	Reduced by a mean of 25 days compared to the prior year

van der Voort et al., 2005 [18]	12 M	Symptoms	WVF: 26 to 4; WNF: 34 to 12 (*p* < 0.05)
		GE	2-hour retention of ILM: 83 to 25%; 4-hour retention of ILM: 38 to 17% (*p* < 0.05)

Lin et al., 2005 [24]	12 M	Symptoms	On PK: TSS: 18.1 to 7.4; off PK: TSS: 17 to 2.6
			On AE: TSS: 19.1 to 9.9; off AE: TSS: 17.7 to 5
		Quality of life	On medication at follow-up: PCS: 21.3 to 33.8; MCS: 36.4 to 50.2
		Days in hospital	50 to 14.9 patients had no admissions
		Medication	Patients requiring PK reduced from 27 to 19; patients requiring AE reduced from 26 to 17

Mason et al., 2005 [33]	20 M	Symptoms	No significance reported
		GE	Rate of emptying: 0.17% to 0.38% per minute (*p* < 0.01)

Lin et al., 2006 [25]	36 M	Symptoms	TSS: 21 to 6 (*p* < 0.05)
		Days in hospital	31 to 5 (*p* < 0.05)
		Medication	Medication use significantly reduced
		Nutrition	Patients requiring nutrition reduced from 15 to 8 (*p* < 0.05)

Anand et al., 2007 [28]	48 M	Symptoms	TSS: 15.6 to 10.9; WVF down to 62%; WNF down to 59%, by 4 years
		GE	2-hour retention of ILM: 55 to 42%; 4-hour retention of ILM: 26 to 17% by 4 Y
		Survival	No significant differences in survival
		Quality of life	IDIOMS score from 16.3 to 10.6 (*p* < 0.05)

Lin et al., 2008 [12]	12 M	Symptoms	TSS decreased from 19.9 to 9.1 (*p* < 0.001)
		GE	2-hour ILM retention: 73% to 63%; 4-hour retention from 46% to 34% (*p* < 0.05); N and V correlate with GE

McKenna et al., 2008 [15]	9.5 M	Symptoms	TSS: 17.1 to 7.7; DG: 16.9 to 5.6 (*p* < 0.05)
		GE	No significant change reported
		Quality of life	No significant change

Brody et al., 2008 [13]	12 M	Symptoms	TSS severity: 19.05 to 14.05; frequency: 20.39 to 15.71
		GE	2-hour retention of ILM: 66% to 50% (*p* < 0.05)

McCallum et al., 2010 [16]	3 M	Symptoms	No significant findings
	12 M	Symptoms	WVF: 19.5 to 4.25; TSS frequency: 18.74 to 11.95; severity: 17.08 to 10.69
		GE	2-hour retention: 76.5 to 51%; 4-hour retention of ILM: 46.5 to 20.5%
		Quality of life	MCS: 29.53 to 36.43; PCS: 33.53 to 40.35
		Days in hospital	40 to 10

McCallum et al., 2011 [29]	56 M	Symptoms	TSS: 19.4 to 9.2; DG: 19.8 to 8.7; IG: 18.6 to 9.7; PSG: 19.1 to 10.9 at 56 M
		GE	2-hour retention: 70 to 66%; 4-hour retention of ILM: 37 to 30% at 56 M

O'Loughlin et al., 2013 [30]	14 M	Symptoms	Total GCSI from 13.4 to 6.6
		GE	Correlation between GCSI and preoperative gastric emptying
		Medication	Significance not reported

McCallum et al., 2013 [19]	12 M	Symptoms	WVF and TSS reduction between ON and OFF not significant
			TSS frequency score from 21.74 ± 1.75 to 13 ± 7.92; *p* < 0.001
			TSS severity score from 18.75 ± 6.34 to 10.26 ± 7.09; *p* < 0.001
			TSS reduction for epigastric pain and burning not significant
		GE	2 hr ILM retention reduction from 63.5% to 49% (*p* ≤ 0.016); 4 hr ILM reduction from 26% to 16.5% (*p* ≤ 0.236)
			Median from 2 to 0 (*p* ≤ 0.06)
		Days in hospital	26.96 to 24.74 (*p* = 0.0768)
		BMI	PCS from 32.66 ± 8.8 to 37.86 ± 13.28 (*p* < 0.043); MCS: 34.11 ± 11.67 to 41.27 ± 12.29 (*p* < 0.001); PF, VT
		Quality of life	SF, MH, and RP *p* < 0.05

Ross et al., 2014 [32]	6 M	Quality of life	Improvement in overall GSRS score, *p* < 0.01
			Median MHC score from 29.15 to 46.6, *p* = 0.01
			Median PHC score from 28.5 to 31.1, *p* = 0.06

Brody et al., 2015 [14]	12 M	Symptoms	TSS functional from 3.2 ± 0.6 to 2.4 ± 0.8; *p* < 0.0001
			TSS pain from 2.8 ± 0.8 to 2.1 ± 0.8; *p* < 0.0001

DG: diabeticgastroparesis; IG: idiopathicgastroparesis; PSG: postsurgicalgastroparesis; CTG: connective tissue disorder.

**Table 3:** Minimum criteria for insertion and reporting outcomes of gastric electrical stimulation (GES).

Assessment	Measure
Quality of life	Short Form 36 (SF-36)

Symptoms	Total Symptom Score (TSS)

Days in hospital	Days in hospital in the last 12
	months

Medication	Is the patient on any prokinetics or
	antiemetics?

Gastric emptying	Standardised scintigraphy analysis of retention of an isotope labelled meal.
